# Design of a robot-assisted system for transforaminal percutaneous endoscopic lumbar surgeries: study protocol

**DOI:** 10.1186/s13018-020-02003-y

**Published:** 2020-10-19

**Authors:** Ning Fan, Shuo Yuan, Peng Du, Wenyi Zhu, Liang Li, Yong Hai, Hui Ding, Guangzhi Wang, Lei Zang

**Affiliations:** 1 Department of Orthopedics, Beijing Chaoyang Hospital, Capital Medical University, Beijing, China; 2 Chaoyang-Tsinghua Digitization & Artificial Intelligence Orthopedic Laboratory, Beijing, China; 3 Department of Biomedical Engineering, School of Medicine, Tsinghua University, Beijing, China

**Keywords:** Medical robotics, Percutaneous endoscopic spinal surgeries, Navigation, Foraminoplasty

## Abstract

**Background:**

Transforaminal percutaneous endoscopic lumbar surgeries (PELS) for lumbar disc herniation and spinal stenosis are growing in popularity. However, there are some problems in the establishment of the working channel and foraminoplasty such as nerve and blood vessel injuries, more radiation exposure, and steeper learning curve. Rapid technological advancements have allowed robotic technology to assist surgeons in improving the accuracy and safety of surgeries. Therefore, the purpose of this study is to develop a robot-assisted system for transforaminal PELS, which can provide navigation and foraminoplasty.

**Methods:**

The robot-assisted system consists of three systems: preoperative planning system, navigation system, and foraminoplasty system. In the preoperative planning system, 3D visualization of the surgical segment and surrounding tissues are realized using the multimodal image fusion technique of computed tomography and magnetic resonance imaging, and the working channel planning is carried out to reduce the risk for injury to vital blood vessels and nerves. In the navigation system, the robot can obtain visual perception ability from a visual receptor and automatically adjust the robotic platform and robot arm to the appropriate positions according to the patient’s position and preoperative plan. In addition, the robot can automatically register the surgical levels through intraoperative fluoroscopy. After that, the robot will provide navigation using the 6 degree-of-freedom (DOF) robot arm according to the preoperative planning system and guide the surgeon to complete the establishment of the working channel. In the foraminoplasty system, according to the foraminoplasty planning in the preoperative planning system, the robot performs foraminoplasty automatically using the high speed burr at the end of the robot arm. The system can provide real-time feedback on the working status of the bur through multi-mode sensors such as multidimensional force, position, and acceleration. Finally, a prototype of the system is constructed and performance tests are conducted.

**Discussion:**

Our study will develop a robot-assisted system to perform transforaminal PELS, and this robot-assisted system can also be used for other percutaneous endoscopic spinal surgeries such as interlaminar PELS and percutaneous endoscopic cervical and thoracic surgeries through further research. The development of this robot-assisted system can be of great significance. First, the robot can improve the accuracy and efficiency of endoscopic spinal surgeries. In addition, it can avoid multiple intraoperative fluoroscopies, minimize exposure to both patients and the surgical staff, shorten the operative time, and improve the learning curve of beginners, which is beneficial to the popularization of percutaneous endoscopic spinal surgeries.

## Background

Percutaneous endoscopic lumbar surgery (PELS) is considered to be a safe and effective kind of minimally invasive surgery (MIS), and is growing in popularity for the management of lumbar disc herniation and spinal stenosis [1, 2]. Compared to traditional open surgery, percutaneous endoscopic lumbar surgery can achieve shorter hospital stay, shorter length of incision, minimal blood loss, and lower visual analog scale (VAS) value back pain [3, 4]. The surgical approaches of PELS include transforaminal and interlaminar approaches, and complete working channel endoscope passage through the intervertebral foramen into the spinal canal from the posterolateral side of the back is called the transforaminal approach [5]. Transforaminal PELS includes three procedures as follows: percutaneous endoscopic lumbar discectomy (PELD) for disc herniation, foraminoplasty for the narrow foramen, and ventral facetectomy for foraminal and lateral recess stenosis [6]. In the surgical technique, the appropriate working channel is a key to achieve success and prevent complications, which are established based on the preoperative imaging and intraoperative fluoroscopy, and the entry point is dictated by many factors such as the size of the patient, dimensions of the facet joints, and the desired location for the tip of the needle in the triangular working zone [7]. However, the landing point and approach angle cannot be accurately adjusted, and the surrounding important tissues are prone to complications such as exiting nerve root and blood vessel injuries [8]. In addition, multiple intraoperative fluoroscopies also increase the radiation exposure of the surgeons and patients. If we can improve the accuracy of the landing point and decrease radiation exposure and complications, it will be beneficial to the popularization of transforaminal PELS.

In the past decades, the use of robotics in surgeries has been successfully employed across many surgical subspecialties, and is considered to set the trend for future surgical development. Rapid technological advancements have allowed robotic technology to assist surgeons in improving the accuracy of surgical maneuvers. Recently, robotics in spinal surgery were predominantly designed as navigational devices for pedicle screw insertion, such as the Mazor X (Medtronic and Mazor Robotics, Medtronic, Minneapolis, MN, USA), ROSA (Med Tech Surgical, Inc., Newark, NJ, USA), and Excelsius GPS (Globus Medical, Inc., Audubon, PA, USA) [9–11]. Multiple studies have reported early experiences with robot-assisted pedicle screw placement and the outcomes are promising [12–14]. Increasingly, researchers have already focused on the combination of MIS and robotic navigation to improve the efficiency and accuracy of spinal surgeries [15, 16]. However, application of robotic technology in percutaneous endoscopic spinal surgery has not been reported up to now.

On the basis of the problems in the application of transforaminal PELS and the development of robotic technology, a safe and effective robot-assisted system for transforaminal PELS must be developed. Therefore, the purpose of the present study is to develop a robot-assisted system for transforaminal PELS. This robot-assisted system can provide navigation, foraminoplasty, and even ventral facetectomy automatically by the 6 degree-of-freedom (DOF) robot arm according to the preoperative planning, and then the surgeon performs further procedures such as discectomy and neural decompression. With a short learning curve and low complication rate, we suppose this robot-assisted system can also reduce the use of intraoperative fluoroscopy, the operative times, and the length of hospital stay.

## Methods

**Fig. 1:**
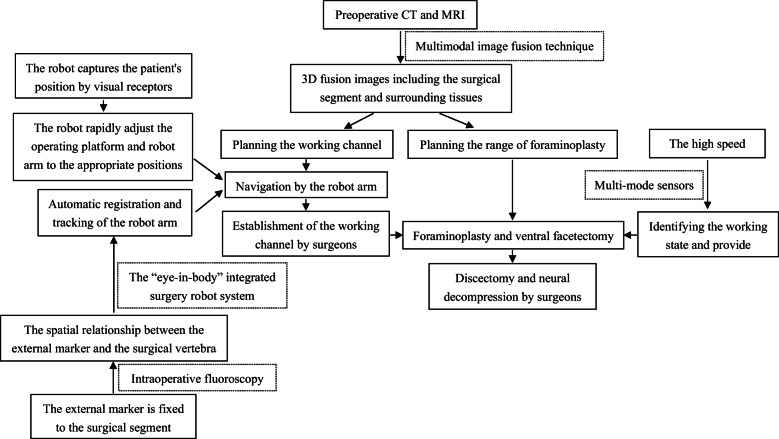
Flow chart shows the processes of development and operation of the system

The robot-assisted system for transforaminal PELS mainly consists of three systems: preoperative planning system, navigation system, and foraminoplasty system. Figure 1 shows the processes of development and operation of this system.

### Preoperative planning system

The surgeon inputs the patient’s preoperative computed tomography (CT) and magnetic resonance imaging (MRI) data into the preoperative planning system, and then this system constructs fusion images using the multimodal image fusion technique. The fusion images including the vertebra, intervertebral disc, nerves, blood vessels, ligamentum flavum, spinal cord, and spinal dura can be displayed on a 2D displayer or 3D using Microsoft HoloLens head-mounted hybrid reality display technology. In this system, the surgeon can clearly observe the anatomical structures of the surgical segment and surrounding tissues preoperatively. In the preoperative planning system, the working channel planning is reasonably carried out for the navigation system using the mouse or keyboard device to make sure the landing point is close to the target and complications are prevented. In addition, the surgeon can mark the range of foraminoplasty for the foraminoplasty system. Our team has made some progress on multimodal image fusion of CT and MRI which is the key for the success of this system.

### Navigation system

**Fig. 2:**
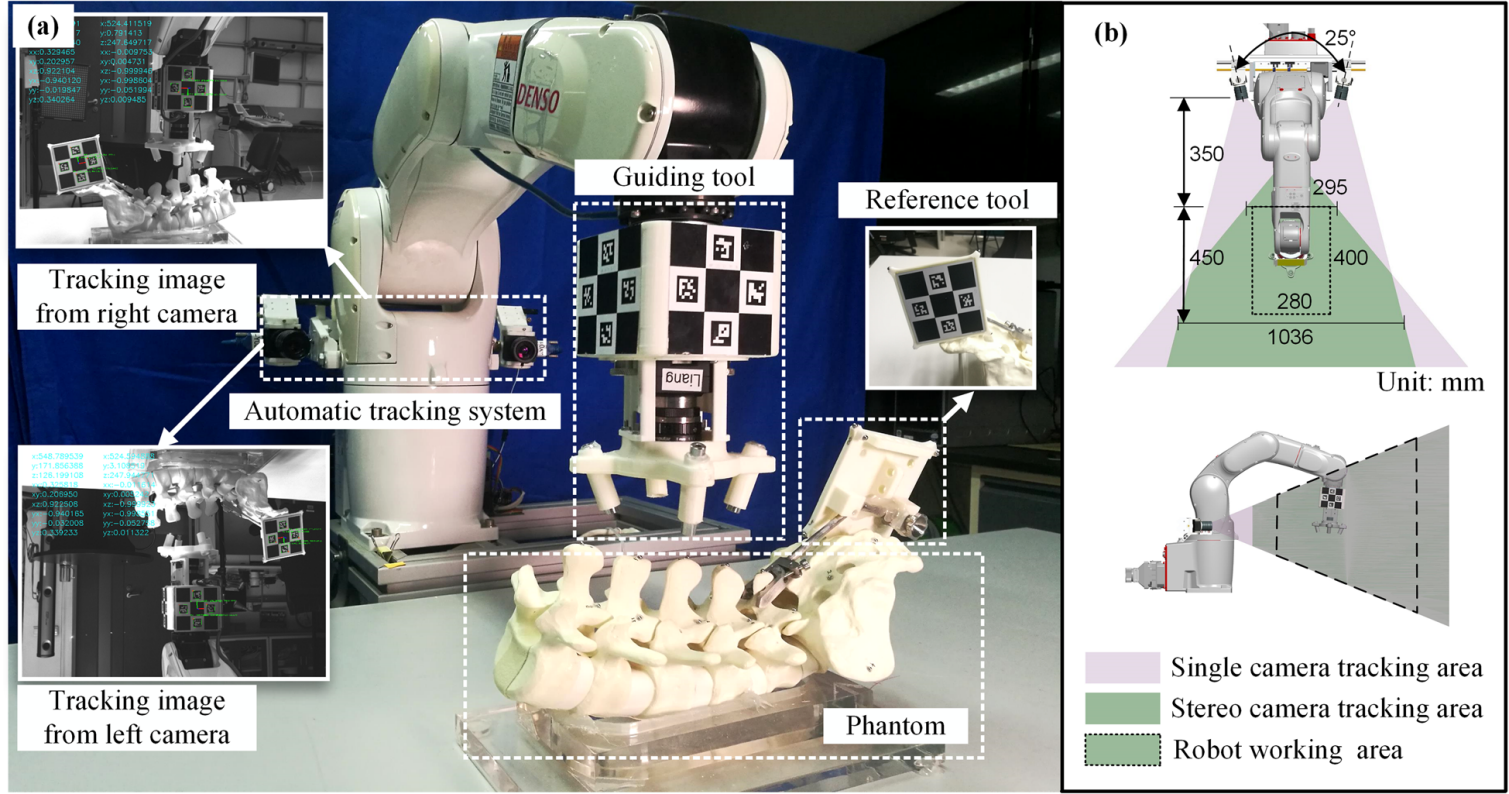
Prototype system overview. **a** Prototype robot system. **b** Tracking system field of view and robot working range

Our team has carried out lots of research on robot navigation and proposed novel “eye-in-body” integrated surgery robotic system [17]. The prototype system (Fig. 2a) has been built using a robot arm (VS060A3, Denso Co. Ltd., Japan), a motion tracking system with two cameras (BFLY-U3-28S4C, FLIR Integrated Imaging Solutions, Inc, Canada), a stepper motor (42BYGH47-1684B-ZK6, Liko Inc, China), and two position sensors (EE-SX672WR, Omron Co. Ltd, Japan). Each camera has a resolution of 1928 × 1448 and lens focal length of 8 mm. Connecting parts are manufactured by 3D printing. The robot controller software is built using Qt, OpenCV, Eigen, and other software packages. The relationship of the tracking system field of view and robot working range is shown in Fig. 2b. This prototype system was mainly designed for the navigation of pedicle screw implantation. This prototype system can perform stereotactic surgery without the intraoperative hand-eye calibration and manual registration, and can achieve an acceptable position and orientation accuracy while tolerating the errors in the hand-eye coordinate transformation error and the robot kinematics model error.

Based on previous studies conducted by our team, we will develop a new robotic navigation system for transforaminal PELS. Firstly, the surgeon fixes the external marker (which can be developed in C-arm fluoroscopy as well as in the visual system) to the surgical segment or adjacent segments after the patient is placed prone on a radiolucent table. Next, the C-arm is used to take anterior-posterior and lateral radiographic images of the lumbar surgical areas. The navigation system can capture the patient’s position and the external marker through visual receptors. According to the external marker and preoperative planning, the robot will automatically adjust the robotic platform and robot arm to the appropriate positions. The above technologies will ensure that the robot arm can work at a small robot working range, has enough flexibility to complete the surgical operations, and realize the rapid placement of the robotic platform and robot arm.

After the robot’s placement, the robot will proceed with automatic registration and tracking of the robot arm. We will use the “eye-in-body” integrated surgery robot system similarly. The position information obtained by the visual system can be directly mapped to the robotic arm, which provides great convenience for automatic registration and tracking of the robot arm. The robot can perform automatic registration of the surgical vertebra by establishing the spatial relationship between the external marker and the surgical segment using intraoperative fluoroscopy. After that, the robot will realize the navigation by the robot arm according to the working channel planning and guide the surgeon to complete the establishment of the working channel.

In addition, as transforaminal PELS is performed under local anesthesia, it is possible that the lumbar vertebra moves, caused by the patient’s movement after vertebral registration during the surgery. To solve this problem, the robot is designed to accurately track the external marker using the visual system. The robot has already established a spatial relationship between the external marker and the surgical target, and the robot can automatically track the moving surgical target through the localization of the external marker intraoperatively. In this way, a fast and highly automatic robot-assisted navigation system can be built to improve the accuracy of the landing point and prevent complications.

### Foraminoplasty system

With the help of the navigation system, the surgeon establishes the working channel. However, if the surgeon inserts the cannula into the narrow foramen, the cannula may compress the exiting nerve root, causing postoperative dysesthesia. To prevent this complication, the surgeon needs to perform foraminoplasty to enlarge the narrow intervertebral foramen, especially in patients with foraminal stenosis. In addition, if the patient is diagnosed with lateral recess stenosis, the procedure of lumbar ventral facetectomy also needs to be performed simultaneously. Therefore, according to the range of bone resection provided by the preoperative planning system, the robot can perform bone resection automatically using a high-speed burr at the end of the 6 DOF robot arm.

However, it is difficult to identify the working status of the high-speed burr, such as the range and degree of bone resection. To solve this problem, we will install a force sensor, acceleration sensor, space position tracking marker, and other sensor devices at the end of the robot arm. Through multi-mode sensors such as multidimensional force, position, and acceleration, the robot can accurately identify the working state of the high-speed burr and provide feedback to the surgeon. Furthermore, through large-scale emulational and cadaveric tests, various signals such as contact force, tool position, and vertebral vibration will be collected during the working process of bone resection. On this basis, an intelligent algorithm will be constructed to establish the quantitative relationship between the sensor signals and the working state (bone contact state, soft tissue contact state, critical state, etc.) of the foraminoplasty system. This system can provide real-time status monitoring and status feedback to the surgeon. The system can realize the status perception and discrimination of surgical instruments in the process of bone resection, ensure the accuracy of foraminoplasty, and improve the safety and effectiveness of the surgical procedure.

### Prototype tests

Prototype of the system has been constructed and performance tests are being conducted in simulations and cadavers. In simulation experiments, we will perform the transforaminal PELS. First, lumbar CT and MRI scans are performed to establish multimodal fusion images, and preoperative planning is carried out in the preoperative planning system. The prototype automatically completes the rapid placement and registration of the surgical segment. The operator establishes a working channel with the navigation of the 6 DOF robot arm, and then the robot completes the foraminoplasty using the high speed burr at the end of the robot arm. After the above operations, we will perform dissection of the surgical segment to evaluate the outcome of foraminoplasty. Furthermore, we shall select fresh thawed cadavers for cadaveric tests, and set up experimental and control groups. In the experimental group, we will perform the procedures of navigation and foraminoplasty with the assistance of the prototype. However, two surgeons who performed 500 PELS in the past 5 years will perform the above procedures without the assistance of the prototype in the control group. When completing the operations, the advantages of the prototype will be evaluated by comparing the outcomes of foraminoplasty, operative time, and radiation exposure between the two groups.

## Discussion

The present study will develop a robot-assisted system for transforaminal PELS, which mainly consists of three systems: preoperative planning system, navigation system, and foraminoplasty system. This robot-assisted system can provide navigation, foraminoplasty, and even ventral facetectomy automatically by the 6 DOF robot arm, according to the preoperative planning system. The development of a robot-assisted system for transforaminal PELS will be of great significance. First, the system can improve the surgical accuracy and efficiency. In addition, it can avoid multiple intraoperative fluoroscopies, minimize exposure to both patientsand surgeons, shorten the operative times, and improve the learning curve of beginners, which is beneficial to the popularization of endoscopic spinal surgeries.

The accuracy of pedicle screw placement is a major concern for spinal surgeons. Although there is a low morbidity of complications, a misplaced screw can result in neurovascular damage, dural tearing, or visceral involvement [18]. Therefore, robot-assisted techniques are designed to help spinal surgeons with more precision and convenience of pedicle screw placement [14–16]. Gao et al. performed a meta-analysis including 6 studies involving 158 patients (688 pedicle screws) in a robot-assisted group and 148 patients (672 pedicle screws) in a freehand group, and showed that the robot-assisted technique was associated with equivalent accuracy rate of pedicle screw implantation, fewer proximal facet joint violations, and less intraoperative radiation exposure, but longer surgical duration than that with the freehand technique [12]. However, a recent meta-analysis of 9 randomized controlled trials with 696 patients, evaluating the accuracy of pedicle screw placement with robot-assisted techniques versus conventional freehand techniques, showed a significant increase in accuracy when using the robot-assisted technique [13]. Spinal robots can provide precise guidance to the areas that are appropriately imaged and registered. Recently, Medtronic announced that they had developed a new robotics platform that combined their intraoperative CT-based spinal navigation system with the Mazor X robotic guidance platform. This robotics platform provides real-time navigated feedback from the instruments to the surgeon, who then uses that information to plan and manually perform the surgical procedure, which adds another layer of safety to robotic pedicle screw instrumentation when deviating from the planned trajectory. Similarly, our robot-assisted system will also provide navigation by the robot arm according to the working channel planning, and guide the surgeon to complete the establishment of the working channel.

In addition, there is no doubt that the spinal robots in the future cannot be limited to assisting surgeons with pedicle screw placement. It should also help to perform different surgical procedures for various diseases. Ponnusamy et al. firstly reported that robotic spinal decompression studied in a porcine model helped to conform that the da Vinci surgical system could perform major non-instrumented procedures of the posterior spine with improved ergonomics and control [19]. Subsequently, Yang et al. reported that use of the da Vinci Surgical System to perform an anterior spinal procedure was shown to be safe and effective in a swine animal model [20]. Currently, the da Vinci robot has been used for anterior lumbar interbody fusion, with a number of case series demonstrating excellent safety profiles in avoiding neurologic, vascular, or ureteral injury during the procedure [21, 22]. However, the da Vinci Surgical System belongs to telesurgical systems in which the robot is under direct control of the surgeon from a remote command station during the operation, and the surgeon’s actions are executed faithfully by the robot in real time. Moreover, the da Vinci system lacks haptic feedback. However, according to the range of bone resection provided by the preoperative planning system, our robot-assisted system can perform bone resection automatically using the high-speed burr at the end of the 6 DOF robot arm. Moreover, the robot can accurately identify the working state of the high-speed burr and provide real-time status monitoring and status feedback to the surgeon using multi-mode sensors such as multidimensional force, position, and acceleration.

The use of the endoscopic technique was restricted to the lumbar disc herniation surgery initially. In recent years, with the development of endoscopic surgical equipment and technology, minimally invasive spinal surgeries can be performed with various endoscopic techniques for the lumbar, cervical, and thoracic regions [23–25]. There has been a growing body of literature that not only confirms the efficacy of the endoscopic technique but also underscores the advantages with respect to less morbidity and safer complications [3, 4, 26–30]. Percutaneous endoscopic spinal surgeries for lumbar, cervical, and thoracic regions categorized according to the surgical approaches are as follows: transforaminal lumbar, interlaminar lumbar, anterior cervical, posterior cervical, posterior thoracic, and posterolateral thoracic [23, 31]. Furthermore, the cervical region has many important anatomical structures such as the trachea, esophagus, carotid artery, and thyroid, and the complications could be serious, such as vascular injury, prevertebral hematoma, swallowing dysfunction, esophageal injury, and nerve injuries [32, 33]. The surgeon needs the landing point to be as close to the target as possible, which makes the learning curve steeper. We suppose the above surgical approaches to the lumbar, cervical and thoracic regions can be accurately set up by our robot-assisted system. In the preoperative planning system, 3D visualization of the anatomical structures of the surgical segment and surrounding tissues are realized using the multimodal image fusion technique. According to the position of spinal stenosis and the types of intervertebral disc herniation in cervical, thoracic, and lumbar regions, the landing point and approach angle are marked to reduce complications. Then, the robot will provide navigation through the robot arm according to the working channel planning, and guide the surgeon to complete the establishment of the working channel. In addition, the high-speed burr can provide bone resection in the working channel.

This robot-assisted system involves biomedical, image processing, medical imaging, robotic dynamic design, and other interdisciplinary and frontier technology. Many challenges need to be overcome and problems solved urgently. However, this robot-assisted system will be a great application prospect that can facilitate the transition from minimally invasive orthopedic surgery to the era of precision medicine and artificial intelligence. Furthermore, we think other surgeries such as robot-assisted percutaneous vertebroplasty, robot-assisted osteotomy, and robot-assisted discectomy should also be considered as the research direction.

## Data Availability

The datasets used and/or analyzed during the current study are available from the corresponding author on reasonable request.

## Abbreviations

PELS: Transforaminal percutaneous endoscopic lumbar surgeries
DOF: Degree-of-freedom
MIS: Minimally invasive surgery
VAS: Visual analog scale
PELD: Percutaneous endoscopic lumbar discectomy
CT: Computed tomography
MRI: Magnetic resonance imaging
